# Recent infection testing to inform HIV prevention responses and surveillance in a programme context: lessons from implementation within a nationally scaled female sex worker programme in Zimbabwe

**DOI:** 10.1002/jia2.26391

**Published:** 2024-11-26

**Authors:** Harriet S. Jones, Fortunate Machingura, Leah Gaihai, Memory Makamba, Thomas Chanyowedza, Panganai Masvikeni, Edward Matsikire, Primrose Matambanadzo, Sithembile Musemburi, Phillip N. Chida, Jeffery Dirawo, Owen Mugurungi, Sarah Bourdin, Bernadette Hensen, Lucy Platt, Gary Murphy, James R. Hargreaves, Frances M. Cowan, Brian Rice

**Affiliations:** ^1^ Faculty of Public Health and Policy, London School of Hygiene & Tropical Medicine London UK; ^2^ Centre for Sexual Health and HIV/AIDS Research (CeSHHAR) Zimbabwe Harare Zimbabwe; ^3^ AIDS and TB Directorate, Ministry of Health and Child Care Harare Zimbabwe; ^4^ Sexual and Reproductive Health Group, Department of Public Health, Institute of Tropical Medicine Antwerp Belgium; ^5^ Independent Consultant London UK; ^6^ Department of International Public Health Liverpool School of Tropical Medicine Liverpool UK; ^7^ Sheffield Centre for Health and Related Research (SCHARR); School of Medicine and Population Health University of Sheffield Sheffield UK

**Keywords:** Africa, HIV epidemiology, HIV prevention, public health, recency assays, sex workers

## Abstract

**Introduction:**

In the context of key population HIV testing programmes, identifying new HIV acquisitions, tracking incidence, and responding with prevention and treatment interventions will be critical for achieving HIV epidemic control. Laboratory tests for recently acquired HIV used as part of a “recent infection testing algorithm” (RITA), offer a potential tool to support this work. We implemented a RITA for female sex workers (FSWs) in Zimbabwe to explore opportunities and programmatic benefits.

**Methods:**

Between October 2021 and January 2023, recency testing was offered to FSWs attending the Centre for Sexual Health and HIV/AIDS Research (CeSHHAR) Zimbabwe's key populations programme. Dried blood spot (DBS) samples were taken at 86 clinic sites across 10 provinces and Laboratory LAg Avidity and viral load testing conducted. RITA results were analysed and linked to programme data to explore geographical differences and calculate HIV incidence. We describe concurrent efforts in HIV testing for social (social network testing [SNT]) and sexual (index case testing [ICT]) contacts of those testing HIV positive.

**Results:**

Among 24,976 FSWs tested at programme sites, 9.5% (2363/24,976) were confirmed HIV positive. We enrolled 55.5% (1311/2363) of eligible HIV‐positive FSWs to our study, of whom 11.7% (153/1311) were identified as having recently acquired HIV. It took a median of 37 days (IQR 20–67) for samples to be processed. Enrolment rates varied between provinces but the proportion of recently acquired HIV was similar (range: 18.4% to 4.0%). Overall HIV incidence was 3.4 (95% CI 2.7−4.0) per 100py. Where results could be linked to routinely collected data, we found no evidence of a difference in test‐positivity between the ICT and SNT contacts of those with recently acquired compared to those with long‐term HIV.

**Conclusions:**

Implementation of a RITA was possible within a nationally scaled sex worker programme, and while challenging to implement, can provide an understanding of transmission dynamics and HIV incidence in this context. Sub‐optimal recruitment and data linkage limited the interpretation of our findings and opportunities for strategic gains though focusing on HIV prevention efforts.

## 1 INTRODUCTION

Data on HIV transmission dynamics can inform HIV prevention and treatment programmes and enable tracking of newly acquired HIV over time to assess the impact of control measures. Among female sex workers (FSWs), who are at high risk of HIV acquisition [1, 2], understanding transmission is critical to improve service delivery and achieve and sustain epidemic control [3]. One approach is recent HIV acquisition surveillance.

HIV‐1 Limiting Antigen Avidity Enzyme Immunoassay (LAg‐Avidity‐EIA) can distinguish recently acquired from long‐standing HIV when combined in a recent infection testing algorithm (RITA) to reduce false positive results. When conducted as part of a RITA, recency testing can identify those who are likely to have recently acquired HIV and help define where current transmission is occurring [4, 5, 6]. The 2022 UNAIDS/WHO guidelines on recency assays highlight potential bias in interpreting results from non‐randomly selected populations accessing HIV testing services [7]. More research is needed to inform implementation and interpretation in these settings [8].

The Centre for Sexual Health and HIV/AIDS Research (CeSHHAR) Key Populations (KP) Programme in Zimbabwe provides free sexual and reproductive health services to women who sell sex. Since its initiation in 2009, programme coverage has increased and those attending services are likely to be representative of FSWs in Zimbabwe [9]. We implemented a RITA across the programme between October 2021 and January 2023. Here, we present the findings of RITA implementation, estimate HIV incidence, and explore the opportunities and strategic benefits of identifying recently acquired HIV in the context of a national FSW programme.

## 2 METHODS

### 2.1 Study setting

CeSHHAR's KP Programme delivers free sexual and reproductive health services across Zimbabwe. The programme predominantly serves cisgender women and girls aged at least 16 years old who sell sex, operating in 86 sites across all 10 provinces in Zimbabwe during the study period. Sites are static clinics delivering services 5 days a week and drop‐in centres and mobile clinics delivering services at least 1 day a week in urban, rural and highway locations. Community outreach services are delivered at each site by FSW peer educators known as microplanners [1, 10].

HIV testing is offered at a first clinic visit to women who are HIV negative or unaware of their HIV status and to HIV‐negative women revisiting a clinic who have not tested in the previous 6 months. In line with Zimbabwe's national HIV testing algorithm, screening is performed with Determine HIV‐1/2 (Abbott Diagnostics, Tokyo, Japan) antibody testing or OraQuick self‐test kits. Confirmatory antibody testing is performed with SD Bioline HIV‐1/2 (Abbott Diagnostics). Pre‐exposure prophylaxis (PrEP) is offered to FSWs testing HIV negative. All women testing positive and not on antiretroviral therapy (ART) are either initiated on, or referred for, treatment as part of Zimbabwe's national HIV treatment programme. Index case testing (ICT) and social network testing (SNT) are conducted for all consenting FSWs testing HIV positive [11, 12]. Through tailored discussions, clinic staff record contact details for all recent sexual partners for ICT and for friends and acquaintances engaged in sex work for SNT. Where consent is obtained, staff identify and offer voluntary HIV testing to individual contacts, or conduct wider testing of known contacts and other individuals in the same location where recent HIV acquisition is identified or direct contact is not considered safe.

### 2.2 Study enrolment

On 5th October 2021, a RITA was introduced as part of our study across the programme. All FSWs testing newly HIV positive were eligible for enrolment if they were 18 years and older and received a confirmatory HIV‐positive result. Those with an indeterminate HIV test result, a history of testing HIV positive >1 year ago or on ART were excluded. Informed consent was sought from all eligible women. Sample size was calculated based on an estimated annual HIV incidence of 6%−10% and 25,000 FSWs undergoing HIV testing in the programme, with 10% test‐positivity, of which 10% would test RITA positive.

A dried blood spot (DBS) sample was collected for each consenting woman. Samples were stored at clinic sites at room temperature in gas‐impermeable plastic bags with desiccant sachets to keep them dry. Samples were collected every 14 days by a designated study driver and delivered to the Flow Cytometry Laboratory in Harare. Clinic staff informed the study coordinator when a new participant was enrolled to arrange and track sample delivery to the laboratory. Laboratory results were shared with relevant programme staff and, in some sites, wider ICT and SNT activities were conducted in response to the identification of recent HIV acquisition. In line with UNAIDS/WHO guidance, laboratory results were not returned to study participants [7].

### 2.3 Laboratory procedures

DBS samples were tested using the Maxim HIV‐1 Limiting Antigen Avidity (LAg Avidity) Enzyme Immunoassay (EIA) following the product insert (DBS KIT CAT NO. 92003) [13]. After initial testing, samples with a normalized optical density (ODn) less than 0.4 underwent serology confirmation with an HIV‐1 ELISA test to confirm an HIV‐positive result. Samples with an ODn >2.0 were immediately classified as long‐term HIV. Those with an ODn ≤2.0 underwent retesting in triplicate from a fresh dilution of the specimen to confirm the results. Final determination of recently acquired or long‐standing HIV for samples with a screening ODn of ≤2.0 was based on the median ODn value of the three retests. A final interpretation of recently acquired HIV required an ODn of ≤1.5 and an ODn of >1.5 for long‐standing HIV. A laboratory specialist examined the relationship between the screening and confirmatory results to determine a final classification, undertaking further testing if necessary.

Viral load (VL) testing was performed with a NucliSens assay on samples with an ODn ≤1.5. Samples with an ODn ≤1.5 and a VL <1000 copies/ml denoted long‐term HIV, and those with an ODn ≤1.5 and a VL ≥1000 copies/ml were classified as recently acquired HIV. Based on resources and the number of samples expected during the study, DBS testing was conducted when 85 samples reached the laboratory. Laboratory staff were trained in specimen handling and testing prior to implementation. Results were validated via the Maxim spreadsheet and external technical support was provided for each testing run.

### 2.4 Data collection

All women accessing CeSHHAR's KP services are assigned a unique identification number on first contact with the programme. Programme data are routinely collected on demographic and clinic visit characteristics and centrally held in an anonymized DHIS2 database. Study participants were assigned an additional unique identification code and data were collected on enrolment site and sample collection date. A centrally held database was kept with anonymized enrolment details and updated when RITA results were returned from the laboratory. For every woman testing newly HIV positive in the programme, data are also routinely collected on their ICT and SNT contacts, the number of contacts reached and tested and the number of those tests that are HIV positive. These data are held in Ministry of Health and Childcare (MoHCC) registers and were anonymized and electronically captured for the period of study implementation. Study and programme data were linked using a separate register holding individual study identification codes and unique programme identification numbers for each participant. RITA study data were incorporated with other programmatic data, and an anonymized data set was generated for analysis.

### 2.5 Data analysis

We firstly present the RITA results, showing sample classification overall and at province level. Using the number of women tested by the programme during study implementation, we estimated HIV incidence among programme attenders [14]. The calculation includes all those at risk of recently acquired HIV, using the number testing HIV negative and recently acquiring HIV; as well as the proportion of FSWs testing HIV positive and enrolled in the study. For our main analysis, we used a mean duration of recently acquired HIV (MDRI) of 130 days and a false recency rate (FRR) of 0.2%. We also calculated incidence using an MDRI of 161 days and an FRR of 0.2% in line with the updated test product insert [13]. For provinces enrolling ≥70% of HIV‐positive FSWs in the study, we calculated province‐specific incidence. Lastly, we describe the results of concurrent efforts to offer HIV testing to sexual and social contacts of FSWs testing HIV positive. We obtained unique identification data to retrospectively link 250 individual FSWs enrolled in our study to their programmatic ICT and SNT data to explore the number of HIV‐positive contacts identified for FSWs with recently acquired and long‐term HIV.

### 2.6 Ethics

Ethical approval was granted by the Medical Research Council of Zimbabwe (MRCZ/A/2244) and the London School of Hygiene & Tropical Medicine (14542 ‑ 1).

## 3 RESULTS

Between 5th October 2021 and 10th January 2023, 24,976 individual FSWs HIV tested at CeSHHAR clinics. Overall, 9.5% (2363/24,976) were newly tested HIV positive, of whom 55.5% (1311/2363) gave consent for a DBS sample to be taken and were enrolled in the study. Study enrolment varied nationally, from 92.2% (188/204) in Bulawayo to 29.4% (25/85) in Matabeleland North (Table 1). FSWs testing HIV positive during the study period had a median age of 30 (IQR 24–36; data for 1374/2366); those enrolled in the study had a median age of 27 (IQR 23−24).

**Table 1:** HIV testing, RITA study enrolment and results by province in Zimbabwe

Province	FSWs tested	HIV positive	%	RITA enrolled	%	Recent HIV	%	95% CI
Bulawayo	2357	204	9%	188	92%	17	9.0%	(5.4%−14.1%)
Harare	3313	378	11%	310	82%	33	10.6%	(7.4%−14.6%)
Manicaland	7124	482	7%	194	40%	18	9.3%	(5.6%−14.3%)
Mashonaland Central	1358	61	4%	34	56%	3	8.8%	(1.9%−23.7%)
Mashonaland East	446	107	24%	47	44%	5	10.6%	(3.5%−23.1%)
Mashonaland West	3222	480	15%	183	38%	32	17.5%	(12.3%−23.8%)
Masvingo	2214	197	9%	108	55%	14	13.0%	(7.3%−20.8%)
Matabeleland North	1025	85	8%	25	29%	1	4.0%	(0.1%−20.4%)
Matabeleland South	2216	246	11%	172	70%	21	12.2%	(7.7%−18.1%)
Midlands	1695	119	7%	49	41%	9	18.4%	(8.8%−32.0%)
Missing	6	4	−	1	−	0		−
Total	24,976	2363	9%	1311	55%	153	11.7%	(10.0%−13.5%)

Abbreviations: FSWs, female sex workers; RITA, recent infection testing algorithm.

**Figure 1:**
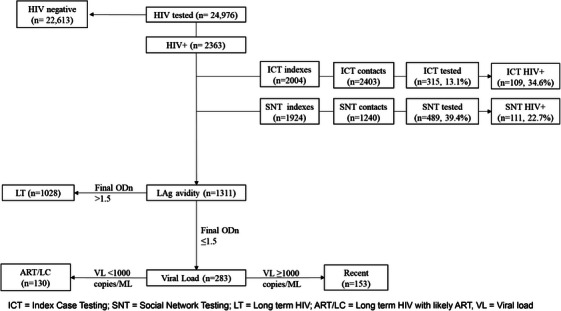
Recent infection testing algorithm flow diagram.

DBS samples took a median of 37 days (IQR 20–67) to reach the Harare laboratory and be processed (data from 1186 samples). No samples were rejected. LAg Avidity test results were obtained for 1311 confirmed HIV‐positive women of which 89 had an ODn threshold <0.4 and underwent serology testing. 1028/1311 samples had a final ODn of >1.5 and were classified as long‐term HIV. The remaining 283/1311 samples had a final ODn ≤1.5 and underwent VL testing, of which 130 had a VL <1000 copies/ml and were also classified as long‐term HIV, with the likelihood of being a woman on ART. The remaining 153/1311 (11.7%, 95% CI 10.0−13.5) samples had a VL ≥1000 copies/ml and were classified as recently acquired HIV (Figure 1). The proportion of recently acquired HIV ranged from 18.4% (9/49; 95% CI 8.8−32.0) in Midlands to 4.0% (1/25; 95% CI 0.1−20.4) in Matabeleland North, with overlapping confidence intervals (Table 1).

### 3.1 HIV incidence

During study implementation, 22,610 individual women tested HIV negative in the programme. Using an MDRI of 130 days and an FRR of 0.2%, we calculated an incidence of 3.4 (95% CI 2.7−4.0) per 100py. Applying an MDRI of 161 days [13], gave an incidence of 2.7 (95% CI 2.2−3.2) per 100py. For Harare, we calculated an incidence of 3.8 (95% CI 2.4−5.1) per 100py and for Bulawayo 2.4 (95% CI 1.2−3.5) per 100py (Table 2).

**Table 2:** HIV incidence calculations

Incidence calculation	Incidence/100py (95% CI)	Details
All data MDRI 130 days, FRR 0.2%	3.35 (2.73−3.96)	In line with ZIMPHIA (2020), earlier Zimbabwe calculations
All data MDRI 161 days, FRR 0.2%	2.70 (2.21−3.20)	In line with updated test insert (DBS KIT CAT NO. 92003)
Matabeleland South^*^	4.17 (2.36−5.95)	12.2% recent HIV
Bulawayo^*^	2.35 (1.19−3.50)	9.0% recent HIV
Harare^*^	3.75 (2.42−5.06)	10.6% recent HIV

Abbreviations: FRR, false recency rate; MDRI, mean duration of recent infection.

* Province incidence calculations—MDRI of 130 days and FRR of 0.2%.

### 3.2 Sexual and social network testing

Between October 2021 and January 2023, ICT and SNT were conducted as part of routine programme implementation. Data were collected from March 2022 in static sites, with mobile sites starting data collection later in 2022. Highway sites did not collect these data. During this period, 2004 FSWs (84.8%; 2004/2363) gave details of 2403 sexual contacts (1.2 contacts per FSW), of whom 315 (13.1%) were identified and tested with 109 (34.6%) testing HIV positive. One thousand nine hundred and twenty‐four FSWs (81.4%; 1924/2363) gave details for 1240 social network contacts (0.64 contacts per FSW), of whom 489 (39.4%) were identified and agreed to an HIV test and 111 (22.7%) subsequently tested HIV positive (Figure 1).

We were able to link a sub‐set of 250 FSWs enrolled in the RITA study to their routinely collected ICT and SNT data. In total, we linked 167 FSWs who provided ICT contacts through whom 51 contacts were identified and tested, with nine (17.6%) testing HIV positive. We linked data for 132 FSWs who provided SNT contacts through whom 172 social network contacts were identified and tested and 25 (14.5%) tested HIV positive. Of FSWs linked to their ICT and SNT data, 25.2% (63/250) had recently acquired HIV. Test‐positivity for SNT contacts among FSWs with recently acquired HIV was 10.2% (12/118; 95% CI 5.4−17.1) compared to 24.1% (13/54; 95% CI 13.5−37.6) among those with long‐term HIV. For ICT contacts identified and tested for FSWs with recently acquired HIV, 22.7% (5/22; 95% CI 7.8−45.4;) were HIV positive compared to 13.8% (4/29; 95% CI 3.9−31.7) among those with long‐term HIV. Included in this analysis were data on 49 FSWs (41 with recently acquired HIV), captured as part of enhanced ICT and SNT testing activities in response to the identification of recently acquired HIV through the RITA study. These programme activities yielded a higher number of contacts tested for FSWs with recently acquired compared to those with long‐term HIV.

## 4 DISCUSSION

Our study provides insights into the novel application of laboratory recent infection testing in a national sex worker programme in Zimbabwe. We recruited 55.5% of all newly HIV‐positive women accessing HIV testing services over 14 months, with higher recruitment in urban sites delivering services 5 days a week. We found that 11.7% had recently acquired HIV, with recency between provinces ranging from 18.4% to 4.0%. National differences in study recruitment made it challenging to determine the extent to which province‐level recency was due to implementation differences or real differences in recently acquired HIV. We used individual programme testing data to calculate an HIV incidence of 3.4 per 100py. For a sub‐set of FSWs, we were able to link programmatic ICT and SNT data; finding no statistical evidence of a difference in test‐positivity between ICT and SNT contacts for those with recently acquired compared to those with long‐term HIV.

Our study had several strengths. We present approaches to the implementation and analysis of recency testing that speak to current gaps in knowledge on the use of recency assays in routine HIV testing services [8] and challenges with recency testing in a programmatic context [6]. Our study contributes to the limited understanding of how to interpret findings from recency testing in routine HIV testing services [8] and to knowledge on HIV incidence among FSWs in Southern Africa [15]. We implemented our study in a high testing frequency setting with good programme coverage, speaking to recommendations that it is only in high coverage settings where inferences can be made [7]. In 2017, CeSHHAR's KP programme reached 57% of the estimated 40,000 FSWs in Zimbabwe [1], and with expansion since, will be reaching many more. An earlier analysis of programme testing data showed increased testing frequency among FSWs over time, with 56.7% of tests among women reporting they had tested in the preceding 6 months by 2018–2019 [16], in line with WHO recommendations for testing every 3–6 months for key populations [11].

Our study also had limitations. The national reach of CeSHHAR's KP programme presented challenges for implementation. Training clinic staff across 10 provinces and 86 sites on recruitment, sample storage and data collection required substantial coordination and oversight. Variations in recruitment between sites may have reflected challenges in training rollout across a wide geographical area and study initiation during the Covid‐19 pandemic. As part of a national MoHCC surveillance initiative, there was concurrent implementation of point‐of‐care testing with Asanté HIV‐1 rapid recency assay in programme sites. Asanté implementation and messaging varied between provinces, potentially leading to conflicting information, test refusal and the additional burden on individuals of conducting two recency tests. Sub‐optimal recruitment is likely to have introduced bias in our findings; however, without data on whether non‐enrolment was due to delayed implementation, tests not being offered or test refusal, it is difficult to assess the direction of any bias. It is likely that within clinic sites, non‐enrolled FSWs did not systematically differ from those enrolled, but differences in enrolment between clinics may have introduced bias if recruitment varied between areas with higher or lower HIV transmission. Limitations in data linkage meant we could not fully characterize the women recruited to our study and make comparisons with those not recruited to further explore potential bias.

Turnaround of samples took over a month. Regular sample collection from programme sites was reliant on clinic staff informing the study coordinator on the day the sample was taken. Delays were possible at sites with service delivery only 1 day a week or with high client‐to‐staff ratios. At the laboratory, samples were stored until the minimum number for batch testing was reached so they were not all tested on arrival. Dual data collection systems with different individual identification numbers meant the integration of study data into routine systems was not always possible and full datasets were not always available. Individual‐level ICT and SNT data were electronically captured from paper registers for this study; however, data were not collected for the entire period of implementation or at highway sites, and linkage was not always possible. The small proportion of linked data limits the extent to which we can draw conclusions from our findings.

We identified a similar proportion of recently acquired HIV to the 10.5% (33/313) found in an earlier pilot in CeSHHAR Zimbabwe's large static programme sites [4, 5, 6]. We identified a higher proportion of recent positive tests than the 6% (18/306) found in a 2021 respondent driven sampling (RDS) survey among FSWs in Zimbabwe [17], and the 1.6% (95% CI 0.9–2.4) among FSWs surveyed in South Africa in 2019 [18]. Our study was conducted in the context of routine service delivery, where testing practices will influence the identification of recent HIV. FSW testing may not be driven by a specific HIV risk exposure if the risk is considered constant. Testing for PrEP may increase testing frequency, but periods of disengagement from sex work may result in disengagement from HIV testing services. An earlier analysis of CeSHHAR's KP programme data showed higher HIV test‐positivity among first‐time testers (13.1% compared to 2.9% among repeat testers) where testing history is less certain and there is a higher potential of a previous diagnosis [16]. An analysis of health records in South Africa between 2017 and 2018 identified that 51.3% of HIV‐positive tests were among individuals retesting [19].

Any national variation in recency was hard to determine and may in part reflect differences in study implementation between sites. Although overall enrolment was lower than the 72% reported in the earlier Zimbabwe pilot [4], enrolment at static sites was higher. Provinces with large urban sites delivering services 5 days a week and regular mobile outreach achieved higher recruitment than provinces with smaller sites, often with lower staffing ratios, delivering services once a week and no static site presence. Despite the reach of CeSHHAR's KP programme, sub‐optimal recruitment in many study sites limits the inferences that can be made about recently acquired HIV among FSWs nationally.

We estimated higher HIV incidence than the 2.1 per 100py, calculated from a RITA in a 2021 RDS survey among FSWs in Zimbabwe [17], but similar to 3.6 (95% CI 2.9–4.6) per 100py from repeat test data in the KP programme between 2018 and 2019 [20]. Estimating seroconversion rates from repeat tests requires subsequent testing with reduced certainty around estimates where there is limited time for individuals to return [20]. We linked programme testing records and calculated incidence using the proportion of recent HIV among those at risk of HIV acquisition, likely to be more indicative of HIV incidence than using only HIV‐positive tests [21]. While we used data from all sites for the period of study implementation, the accuracy of our estimates may have improved by specifying site‐specific implementation periods and varying study enrolment. We used an MDRI of 130 days for our main incidence calculation in line with Zimbabwe's 2020 PHIA survey [22] and previous estimates from FSW RDS surveys [17]. We applied an FRR of 0.2% in contrast to 0 which suggests no false recent results and is unlikely, even with a RITA [23].

Through retrospective linkage of programme ICT and SNT data, we found no statistically meaningful difference in the proportion of HIV‐positive contacts identified through ICT and SNT among FSWs with recently acquired and long‐term HIV, with overlapping confidence intervals between the groups. While programmatic gains from using recency‐informed ICT and SNT are not indicated by our findings, limitations in our data make interpretation challenging. Our findings were likely to be biased by sub‐optimal record linkage and a higher proportion of FSWs with recently acquired HIV in this analysis compared to our main RITA results. In addition, data on wider HIV testing in locations where contacts were identified in response to a RITA‐positive result were easier to link and yielded higher numbers of contacts tested for FSWs with recently acquired HIV compared to those with long‐term HIV; limiting our comparison of the proportion of HIV‐positive tests among those tested between the two groups. A 2021 study in Nigeria identified additional HIV‐positive cases through ICT among recent positives, although did not make comparisons between participants with recently acquired or long‐term HIV [24]. A 2018 study of recency testing in routine services in Kenya found a greater proportion of HIV‐positive ICT contacts previously unaware of their HIV status among participants with recently acquired HIV, although overall similar numbers of HIV‐positive contacts for those with recent and long‐term HIV [6, 25]. As with our study, neither provided conclusive evidence of strategic gains from focusing ICT resources among those with recently acquired HIV.

With documented challenges in implementing and interpreting rapid recency assays in field settings [4, 26], our study explored whether laboratory‐based recency testing could provide an alternative. While we demonstrate that DBS sample collection, storage, transport and laboratory analysis were feasible, implementing a RITA across a nationally scaled programme requires substantial resources. The turnaround time from sample collection to laboratory processing and linkage of results has implications for informing a programmatic response. Operationalizing recency testing requires data systems that can link HIV test and recency results back to clinics, while ensuring confidentiality. While UNAIDS/WHO do not recommend the use of recency assays for clinical management [7], we hypothesized that recency assays could be useful for the identification of geographic clusters of recent HIV, and inform more targeted approaches to ICT and SNT, which when unguided takes substantial resources to identify a relatively small number of new HIV acquisitions. Our findings were unable to support this, with little variation in recent HIV between provinces or evidence of higher test‐positivity for SNT or ICT among FSWs with recently acquired HIV. Further exploration of recency testing to guide ICT and SNT would be needed to draw any robust conclusions. We were able to estimate HIV incidence which could have advantages over the estimation of seroconversion rates through individual test linkage for repeat testers, providing more up‐to‐date information.

## 5 CONCLUSIONS

RITAs are challenging to implement in a programmatic context and integrate within existing data systems, limiting the interpretation of findings and opportunities for a programmatic response. Our study demonstrates that implementation within a nationally scaled sex worker programme is possible, and while substantial programme capacity is required, understanding transmission dynamics through a RITA could support efforts to estimate HIV incidence.

## COMPETING INTERESTS

The authors declare no conflicts of interest.

## AUTHORS’ CONTRIBUTIONS

FMC, BR and FM conceived and planned the RITA study. HSJ conducted the analysis and wrote the manuscript with input from BR, FMC, GM, FM, PM and JRH. FM is the Key Populations research director at CeSHHAR and, with LG, MM, PM and EM, implemented the study. FMC and BR provided technical guidance on implementation. GM provided technical guidance on laboratory procedures. PM is the Key Populations programme director at CeSHHAR, overseeing the implementation of the Key Populations programme. JD, SM, PNC and TC oversaw and managed the programme and study data. Co‐authors reviewed the manuscript and provided input on analysis, interpretation of results and writing. All authors have approved the final manuscript.

## FUNDING

CeSHHAR's Key Populations programme has been funded by the UN Population Fund (through Zimbabwe's Integrated Support Fund funded by UK Department for International Development, Irish Aid, and Swedish International Development Cooperation Agency), Deutsche Gesellschaft für Internationale Zusammenarbeit, the Bill & Melinda Gates Foundation, The Global Fund to Fight AIDS, Tuberculosis and Malaria, the US President's Emergency Plan for AIDS Relief, the US Agency for International Development, and the Elton John AIDS Foundation. HSJ was funded by the Medical Research Council's London Intercollegiate Doctoral Training Partnership. FMC and JRH were partly funded by the Wellcome Trust (214280/Z/18/Z). JRH, FMC, BR, LP, and HSJ were members of the Measurement and Surveillance of HIV Epidemics Consortium (London School of Hygiene & Tropical Medicine, London, UK), which received a grant from the Bill & Melinda Gates Foundation to develop, test, and implement innovative and efficient methods for routine HIV measurement and surveillance (INV‐007055). The RITA study was supported by the Bill & Melinda Gates Foundation, grant number INV‐007055. Under the grant conditions of the UK Research and Innovation and the Bill & Melinda Gates Foundation, a Creative Commons Attribution 4.0 Generic License (CC BY) has already been assigned to any Author Accepted Manuscript version arising from this submission. The findings here are those of the authors and do not necessarily represent the views or official position of the funding agencies. We acknowledge everyone who has dedicated time to implementing CeSHHAR's Key Populations programme. We thank the women who enrolled in the RITA study and those who visited the Key Populations programme during the study period, all contributing data to this analysis.

## Data Availability

A de‐identified dataset with variables included in this analysis can be made available on request to the Centre for Sexual Health & HIV/AIDS Research Zimbabwe, subject to ethical approval of a proposal.
